# Neurological Manifestations of COVID-19 in Absence of Respiratory Symptoms or Fever

**DOI:** 10.7759/cureus.13887

**Published:** 2021-03-14

**Authors:** Abbas Mohamed, Ahmad S Qureshi, Sarah A Mohamed

**Affiliations:** 1 Laparoscopic Surgery, National Guard Hospital, Al Madinah, SAU; 2 Pulmonology, National Guard Hospital, Al Madinah, SAU; 3 General Surgery, Surgical Department, University Hospital of Wales, Cardiff, GBR

**Keywords:** : (sars-cov-2) /covid-19, neurological manifestations

## Abstract

The novel coronavirus disease 2019 (COVID-19), caused by severe acute respiratory syndrome coronavirus 2 (SARS-CoV-2), was first described in the Hubei region of China and was declared a pandemic by the World Health Organization (WHO) in March 2020.

The clinical presentation of COVID-19 ranges from asymptomatic/mild symptoms to severe illness with associated mortality. The most commonly reported symptoms of COVID-19 are cough, myalgia, and headache. Other well-described features include shortness of breath, fever, sore throat, diarrhea, and smell or taste abnormalities.

Although COVID-19 infection is primarily a disease of the respiratory system affecting lung parenchyma, it can affect multiple organ systems and cause variable extra-pulmonary symptoms. A broad spectrum of neurologic manifestations has been reported in association with SARS-CoV-2 infection, most probably due to different pathogenic pathways. We report a case of a rare presentation of a COVID-19-positive female who presented with slurred speech, dizziness, and left-sided weakness without the other common symptoms of the disease.

## Introduction

Since the outbreak of the novel coronavirus disease 2019 (COVID-19), caused by severe acute respiratory syndrome coronavirus 2 (SARS-CoV-2), in March 2020, there have been various reports of its symptoms and outcomes focusing mainly on respiratory complications. Recently, many reports have been published describing the effects of the virus on the nervous system [1]. It was reported that the infection could be associated with various neurological manifestations, including ischemic stroke, intracerebral hemorrhage, encephalopathy, anosmia, and neuromuscular diseases [2]. The neurological manifestations usually occur in association with the other common manifestations of the disease such as fever, cough, shortness of breath, and other respiratory symptoms but rarely in their absence. We report a case of a 42-year-old COVID-19-positive female who presented with slurred speech, dizziness, and left-sided weakness in the absence of respiratory symptoms or fever.

## Case presentation

A 42-year-old obese female with a BMI of 41 presented to the emergency room with slurred speech, dizziness, and left-sided weakness which started about two hours before presentation. The patient did not report any cough, fever, shortness of breath, loss of smell, or body aches. She had no pre-existing medical conditions and was not taking any medications. She was a non-smoker. There were no sick contacts at home. On examination, she was hemodynamically stable alert, awake, and oriented with oxygen saturation of 98% in room air. Her pulse rate was 85/minute, her blood pressure 130/85 mmHg, and her Glasgow Coma Score was 15/15. In neurological examination, she had slurred speech and 3/5 weakness in the left upper and lower extremity but normal sensations with no neck stiffness. The rest of the neurological examination as well as chest, cardiovascular, abdominal, and musculoskeletal examination was normal. 

**Figure 1:**
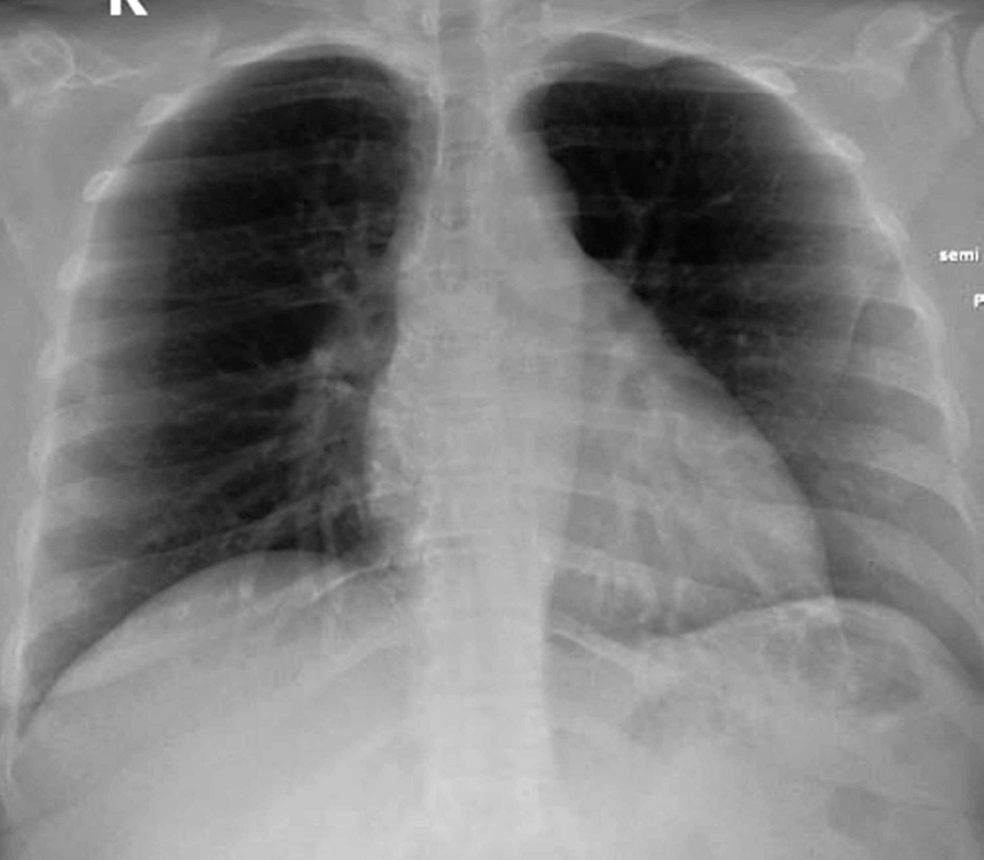
Chest X ray showing no pulmonary consolidation or infilteration.

The laboratory investigations showed hemoglobin of 12.4 gram/L, white blood cells (WBC) 7.2x109, lymphocytes 17% and C-reactive protein 8.7 mg/L (normal value 0.1-4.9 mg/L). Blood culture done on admission was negative for any bacterial growth. All other investigations, including urea and electrolytes, liver function test, D dimer, and coagulation profile were within normal limits. Her chest x-ray was normal without evidence of consolidation or infiltration (Figure 1). 

**Figure 2:**
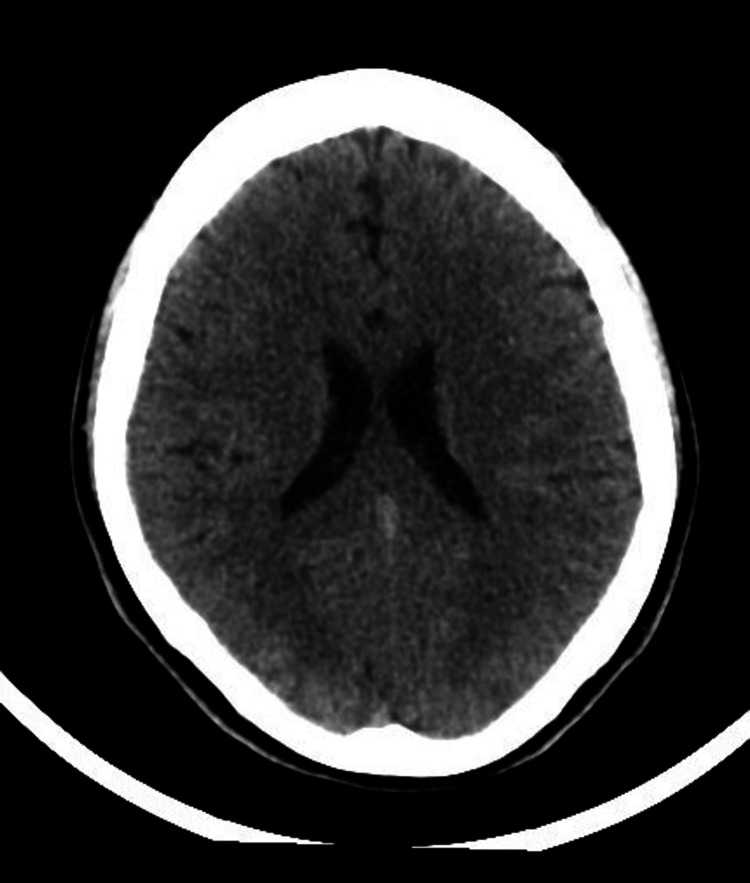
Brain CT scan view showed no evidence of intracranial hemorrhage, or focal mass lesions with the normal appearance of the ventricular system.

**Figure 3:**
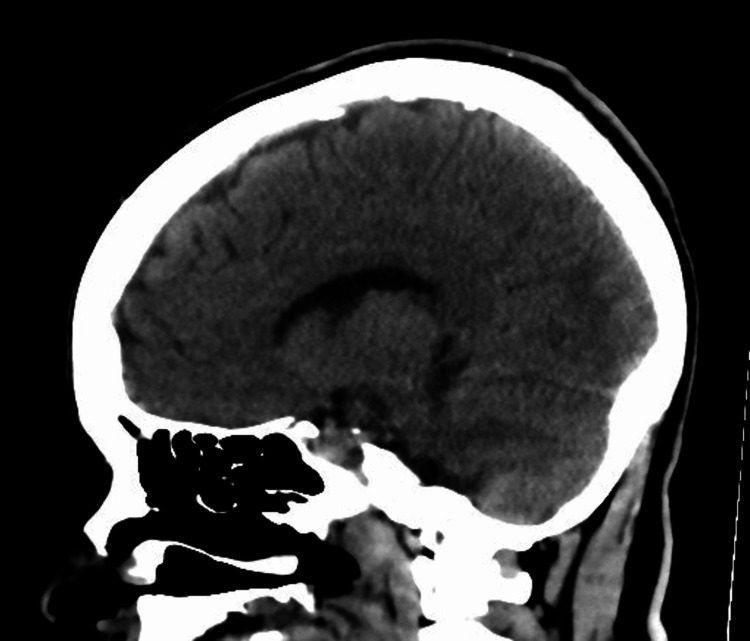
Brain CT scan (sagittal view) showing no evidence of intracranial hemorrhage, or focal mass lesions.

The patient was within the window of tissue plasminogen activator (TPA) for acute stroke and underwent an emergent brain CT scan without contrast which showed no evidence of recent major territorial infarction, intracranial hemorrhage, or focal mass lesions with the normal appearance of the ventricular system and posterior fossa structures (Figures 2, 3).

She was treated with intravenous TPA and was admitted to the intensive care unit (ICU) for monitoring. She did not have any abnormal electrocardiogram (EKG) rhythm while in ICU and had full recovery of her neurological symptoms within few hours without any bleeding complications. She was screened for COVID-19 on admission as per hospital protocol and the result came back later as positive. She was subsequently discharged in good health and advised home isolation.

## Discussion

Since COVID-19 was declared a pandemic by the World Health Organization in March 2020, various reports have been published outlining its various clinical presentations, with a particular focus on respiratory symptoms. However, there is growing evidence of the effects of the virus on the nervous system and its neurological manifestation. Patients infected with COVID-19 may have a wide range of symptoms, ranging from asymptomatic or mild symptoms to severe disease associated with mortality. The most common symptoms are cough, breathlessness, and fever. Other symptoms, such as sore throat, malaise, headache, muscular pain, nausea, vomiting, diarrhea, loss of taste and smell, and respiratory distress have also been described [3].

While COVID-19 is primarily a respiratory disease that affects pulmonary parenchyma, it can also affect multiple organs and cause variable extrapulmonary symptoms. As the COVID-19 pandemic progresses, reports of neurological manifestations are increasing; to date, 901 patients have been reported having neurological symptoms [4]. Because of the emergence of new strains and the continuation of the pandemic, it is expected that 50-80% of the world's population might become infected, which may lead to a significant overall increase in the number of patients with COVID-19 neurological manifestations.

Symptoms and neurological events associated with COVID-19 are rare and may affect three different systems: the central nervous system (CNS), the peripheral nervous system, and the musculoskeletal system [5]. It seems that the virus enters and damages the CNS by systemic hematogenous spread or neuronal retrograde dissemination, however, the precise pathogenesis of COVID-19 in the nervous system remains unclear [6]. Neurological manifestations of COVID-19 range from milder manifestations such as headache, anosmia to serious complications such as seizures, strokes, intracerebral bleeding, encephalopathies, anosmia, and neuromuscular disorders [1]. Recent studies of neurological manifestations have shown an increase in the frequency and variability of neurological outcomes.

A retrospective study that included 222 COVID-19 patients with neurologic manifestations from 46 centers in France reported that the most common neurological manifestations of COVID-19 are encephalopathy (30.2%), acute ischemic cerebrovascular syndrome (25.7%), encephalitis (9.5%), and Guillain-Barre syndrome (6.8%). The study found that the clinical spectrum and outcomes of neurological events associated with COVID-19 infection were broad and heterogeneous, suggesting that the underlying pathogenic processes were different [7].

Another retrospective study conducted in Wuhan, China, involved 221 cases of COVID-19 and revealed that 11 patients (5.0%) had an acute ischemic stroke [8]. Oxley et al. reported five cases of large-blood vessel strokes in patients who tested positive for COVID-19 under the age of 50, two of whom were asymptomatic [9]. Fu et al. reported two cases of COVID-19 pneumonia with acute ischemic stroke in middle-aged patients. Both patients presented with tongue palsy, dysarthria, and limb muscle weakness as initial manifestations of the disease and in the absence of brain CT scan abnormalities [10]. Similarly, Zhai et al. report a case of ischemic stroke (lacunar infarction) in a 79-year-old man who presented with right limbs weakness dysarthria and tongue deviation and was diagnosed later with COVID-19 [11].

Our case presented with slurred speech, dizziness, and left-sided weakness in absence of fever, respiratory symptoms and CT scan evidence of ischemic stroke or intracranial hemorrhage and is one of the rare reported cases of neurological manifestations of COVID-19 infection. Our case report also shows the safe use of TPA in this patient population as well as a unique neurological presentation in the absence of respiratory symptoms. A retrospective study from Wuhan, China, that involved 214 COVID-19-positive patients reported two cases similar to our case that presented with sudden onset of hemiplegia without fever or upper respiratory tract symptom [12].

 

## Conclusions

While COVID-19 is primarily a respiratory disease that affects pulmonary parenchyma, it can also affect multiple organs and cause variable extrapulmonary symptoms. The neurological symptoms and manifestations of COVID-19 are rare and the proportion of patients with neurological manifestations is small compared with that of the respiratory disease. As the pandemic progresses, the reports of neurological manifestations are expected to increase. Clinicians should be familiar with the link between COVID-19 infection and various neurological manifestations for early diagnosis and management. As the neurological manifestations of the disease are variable and can occur in the presence or absence of other symptoms and CT scan evidence of brain insult, we recommend reporting all cases of these manifestations.
